# In triple negative breast tumor cells, PLC-β2 promotes the conversion of CD133^high^ to CD133^low^ phenotype and reduces the CD133-related invasiveness

**DOI:** 10.1186/1476-4598-12-165

**Published:** 2013-12-13

**Authors:** Federica Brugnoli, Silvia Grassilli, Manuela Piazzi, Maria Palomba, Ervin Nika, Alberto Bavelloni, Silvano Capitani, Valeria Bertagnolo

**Affiliations:** 1 Signal Transduction Unit, Section of Anatomy and Histology, Department of Morphology, Surgery and Experimental Medicine, University of Ferrara, Via Fossato di Mortara 70, 44121 Ferrara, Italy; 2 Department of Human Anatomical Sciences, Cellular Signalling Laboratory, University of Bologna, Via Irnerio 48, 40126 Bologna, Italy; 3 Laboratory of Musculoskeletal Cell Biology, Rizzoli Orthopedic Institute, Via di Barbiano 1/10, 40136 Bologna, Italy

**Keywords:** Breast cancer, Phospholipase C-β2 (PLC-β2), CD133, Tumor progression

## Abstract

**Background:**

Beyond its possible correlation with stemness of tumor cells, CD133/prominin1 is considered an important marker in breast cancer, since it correlates with tumor size, metastasis and clinical stage of triple-negative breast cancers (TNBC), to date the highest risk breast neoplasia.

**Methods:**

To study the correlation between the levels of CD133 expression and the biology of breast-derived cells, CD133^low^ and CD133^high^ cell subpopulations isolated from triple negative MDA-MB-231 cells were compared in terms of malignant properties and protein expression.

**Results:**

High expression of CD133 characterizes cells with larger adhesion area, lower proliferation rate and reduced migration speed, indicative of a less undifferentiated phenotype. Conversely, when compared with CD133^low^ cells, CD133^high^ cells show higher invasive capability and increased expression of proteins involved in metastasis and drug-resistance of breast tumors. Among the signalling proteins examined, PLC-β2 expression inversely correlates with the levels of CD133 and has a role in inducing the CD133^high^ cells to CD133^low^ cells conversion, suggesting that, in TNBC cells, the de-regulation of this PLC isoform is responsible of the switch from an early to a mature tumoral phenotype also by reducing the expression of CD133.

**Conclusions:**

Since CD133 plays a role in determining the invasiveness of CD133^high^ cells, it may constitute an attractive target to reduce the metastatic potential of TNBC. In addition, our data showing that the forced up-regulation of PLC-β2 counteracts the invasiveness of CD133-positive MDA-MB-231 cells might contribute to identify unexplored key steps responsible for the TNBC high malignancy, to be considered for potential therapeutic strategies.

## Introduction

Breast cancer represents a heterogeneous group of tumors with different morphology, biology and treatment approach [1]. Triple-negative breast cancers (TNBC), as defined on the basis of immunohistochemistry and for typically being negative for estrogen receptor (ER), progesterone receptor (PR) and HER2, represent approximately 20% of all breast tumors and have a considerable clinical relevance as they primarily affect young women, appear resistant to conventional chemotherapy regimens, have a particularly poor prognosis and a significantly worse clinical outcome than other tumor types [2]. In the management of patients with TNBC, a promising role seems to be played by the observed relationship between the positivity to the glycosylated trans-membrane protein CD133 and shorter disease free and overall survival, suggesting that CD133 expression may be of help in more accurately predicting the aggressive properties of this neoplasia [3]. Although a wide range of studies suggest that CD133-positivity identifies cancer stem cells [4] yet the ability of CD133 to reliably identify breast tumor progenitors is controversial, also due to the use of different antibodies recognizing CD133 splice variants with epitopes of different glycosylation status [5]. A strong correlation between CD133 expression and aggressive cellular behavior, including resistance to chemotherapy and radiotherapy, was also observed in hepatocellular carcinoma [6], colon cancers [7] and malignant gliomas [8,9], indicating that, regardless its role as a marker of stemness of tumor cells, CD133 may constitute a prognosticator for a number of different neoplasia.

A functional role of CD133 in tumors is suggested by the evidence that *in vitro* targeting of CD133 with a specific binding peptide reduced colon and breast tumor cell motility [10] and *in vivo* down-regulation of CD133 severely impaired the capacity of melanoma cells to metastasize [11]. Successful immunotoxin targeting of CD133 in hepatocellular and gastric cancer xenografts has also been reported [6], suggesting that CD133 may be an important cancer therapeutic target. On the contrary, even though recent in vitro data on TNBC correlate CD133 with the inhibitor of cell cycle progression Geminin [12], at present there is no evidence that associates CD133 to intracellular proteins involved in signalling events promoting breast tumor malignancy and very little is known about the regulation of its expression in breast tumor cells [13]. A number of signalling molecules are deregulated in breast neoplasias, including specific isoforms of phosphoinositide-dependent phospholipase C (PLC) that resulted variously involved in proliferation, migration and invasiveness of tumor cells [14-17]. We have demonstrated that PLC-β2 expression strongly correlates with a poor prognosis of patients with breast tumors [18] and that, in breast tumor-derived cells with a triple negative phenotype, this PLC isozyme promotes migration and is necessary to sustain invasion capability [16].

Aim of this work was to elucidate whether CD133 has a role in determining the malignancy-related properties of TNBC-derived cells. The relationship of CD133 expression with proteins known to be de-regulated in breast neoplasias, particularly with PLC-β2, was also investigated.

## Results

### High expression of CD133 characterizes cells with high invasion capability

**Figure 1:**
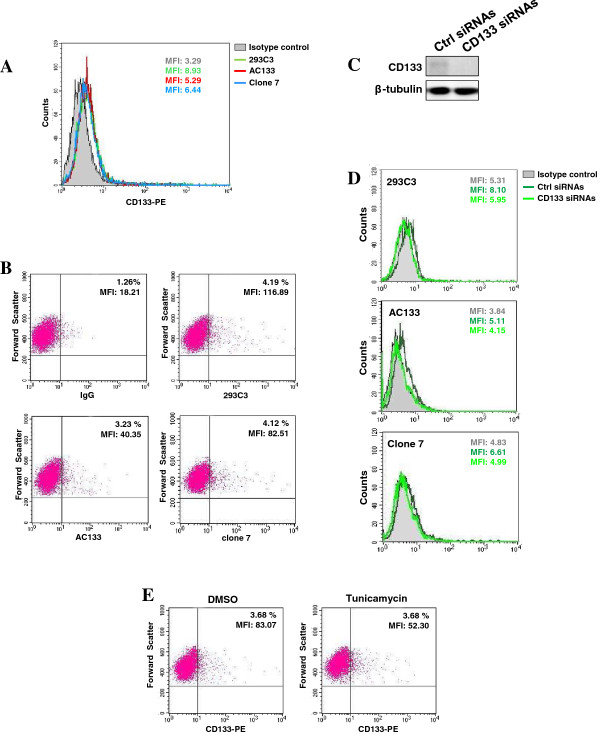
**CD133 expression in MDA**-**MB**-**231 cells. (A)** CD133 surface expression evaluated in MDA-MB-231 cells by means of flow cytometry after staining with CD133/2 (293C3) and CD133/1 (AC133) phycoerythrin conjugated antibodies and with a hybridoma supernatant (clone 7). The expression of each antigen is represented on a frequency distribution histogram (count vs. PE signal). The open histograms, outlined by coloured lines, represent positive staining for CD133 and gray filled histogram shows negative control stained with matched isotype antibody. **(B)** The surface expression of CD133, measured with the indicated antibodies, is presented on a biparametric dot plot. The quadrants are gated to separate positive populations. The percentage of cells expressing high levels of CD133 is indicated at the upper right of each panel, together with their mean fluorescence intensity (MFI). **(C)** Western blot analysis with the anti-CD133 antibody on CD133 immunoprecipitated from MDA-MB-231 cells transfected with non-silencing RNAs (Ctrl siRNAs) or with siRNAs specific for CD133 (CD133 siRNAs). Lysates from the same cells were analyzed for β-tubulin content, as internal control of processed proteins. **(D)** Cytofluorimetrical analysis of CD133 expression performed in MDA-MB-231 cells transfected with non-silencing RNAs (Ctrl siRNAs) or with siRNAs specific for CD133 (CD133 siRNAs). The open histograms, outlined by coloured lines, represent positive staining for CD133 and gray filled histograms show negative controls stained with matched isotype antibody. **(E)** CD133 surface expression measured with PE-conjugated 293C3 antibody in MDA-MB-231 cells cultured in the presence of Tunicamycin for 24 hours and shown as dot plots in which the percentage of positive cells and their mean fluorescence intensity (MFI) are indicated at the upper right. The data are representative of three separate experiments.

MDA-MB-231 cells were subjected to cytofluorimetrical analysis with two commercially available antibodies directed against two different CD133 glycosylated epitopes (293C3 and AC133), and an anti-human CD133 monoclonal antibody able to specifically recognize an unmodified CD133 extracellular domain (clone 7). Immunophenotyping with the three antibodies showed similar results indicating that the entire cell population expresses low levels of CD133 (Figure 1A) and that a small subset of cells (about 2-3%) express CD133 at much higher levels (Figure 1B). The specificity of all the used anti-CD133/antibodies was confirmed by silencing CD133 expression with specific siRNAs (Figure 1C, D). The use of Tunicamycin allowed to confirm that the glycosylation levels of CD133 do not affect the capability of antibodies to identify expressing cells but may influence, as expected, the fluorescence intensity, indicative of the accessibility of the antibody to its specific target epitopes (Figure 1E, Additional file 1: Figure S1).

**Figure 2:**
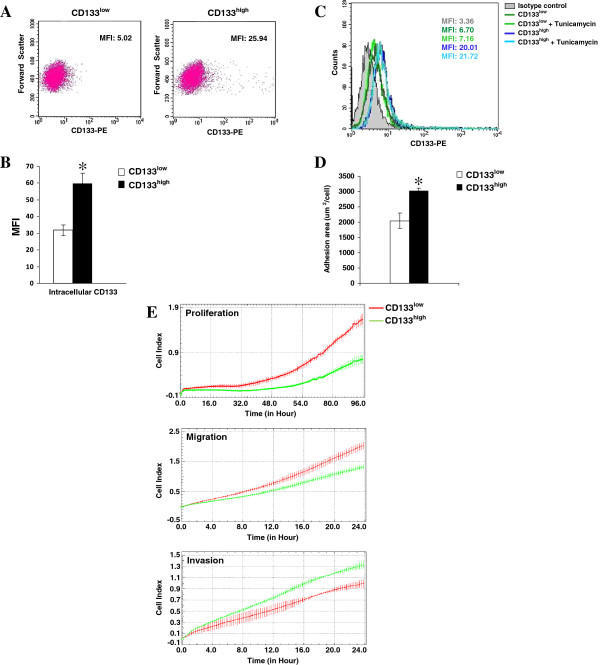
**CD133**-**related phenotype of MDA**-**MB**-**231 cells. (A)** CD133^low^ and CD133^high^ cells, obtained from MDA-MB-231 cells by positive magnetic separation with CD133/1 Micro Beads, were subjected to the cytofluorimetrical evaluation of CD133 expression with PE-conjugated 293C3 antibody, according to the manufacturer instructions. The data are presented on a biparametric dot plot, in which the mean fluorescence intensity (MFI) of the entire population is indicated. **(B)** Intracellular CD133 amount in CD133^low^ and CD133^high^ cells measured with PE-conjugated 293C3 antibody. The MFI of the entire populations were presented as column bar ± SD. The asterisk denotes statistical difference (P < 0.05). **(C)** CD133 surface expression measured by flow cytometry after staining with PE-conjugated 293C3 antibody of CD133^low^ and CD133^high^ cells cultured in the presence of 2.5 μg/ml Tunicamycin for 24 hours. The open histograms, outlined by coloured lines, represent positive staining for CD133 and gray filled histogram shows negative control stained with matched isotype antibody. **(D)** Adhesion area of CD133^low^ and CD133^high^ cells measured with the ImageJ software. The data are the mean of 3 separate experiments ± SD. The asterisk indicates statistically significant difference (*P* < 0.05). **(E)** Dynamic monitoring of proliferation, migration and invasiveness through Matrigel using xCELLigence system for the indicated times of CD133^low^ and CD133^high^ cells. Error bars indicate ± SD. The data are representative of three separate experiments.

Positive immunomagnetic separation of MDA-MB-231 cells with the AC133 antibody generated two sub-populations with significantly different expression levels of CD133. In particular, a CD133^low^ cell population corresponded to about 93% of cells and a CD133^high^ subpopulation, that included the cells with the greatest expression of CD133, accounted for about 7% of cells (Figure 2A). The analysis of intracellular CD133 confirmed the significant difference of CD133 expression shown by the two sub-populations (Figure 2B). In addition, the use of Tunicamycin excluded the possibility that the difference in fluorescence intensity displayed by the two subpopulations depended on variable glycosylation levels of CD133, as shown by the overlapping of the cytometric profiles in the presence or absence of the drug (Figure 2C).

CD133^low^ and CD133^high^ cells were grown in the same standard culture conditions, showing a stable difference in CD133 expression levels up to at least 2 passages in monolayer culture (72 hours).

After 24 hours from separation, CD133^low^ and CD133^high^ cells were evaluated for morphology and subjected to impedance-based xCELLigence Real-Time Cell analysis. Compared to CD133^low^ cells, CD133^high^ cells showed larger adhesion area (Figure 2D) and lower proliferation rate and motility (Figure 2E), suggestive of a less undifferentiated tumoral phenotype. On the contrary, invasiveness measured through Matrigel-coated membranes resulted significantly higher for CD133^high^ cells (Figure 2E).

### High CD133 levels correlate with a peculiar protein expression pattern

To search for specific protein signatures associated to the two cell subsets identified by CD133 quantitative immunophenotyping, CD133^low^ and CD133^high^ cells were subjected to proteomic analysis by performing two dimensional electrophoresis followed by mass spectrometry.

**Figure 3:**
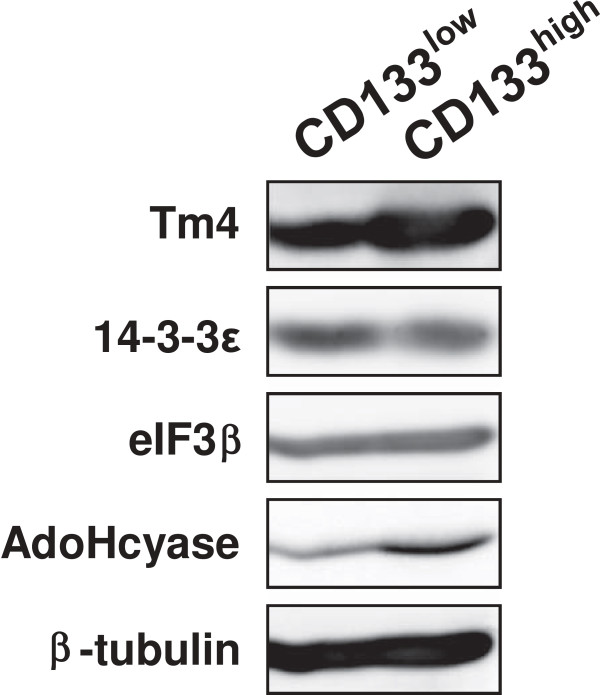
**Protein profiles of CD133**^**low **^**and CD133**^**high **^**sub**-**populations.** Total lysates from CD133^low^ and CD133^high^ cells were subjected to immunochemical analysis with the indicated antibodies. Tubulin was blotted as a control for the equivalence of loaded protein. The data are representative of three separate experiments.

By using the PDQuest software, after removal of saturated and poorly reproducible zones, about 380–420 spots *per gel* were compared to select proteins whose amount showed a significant degree of variability between the two subpopulations expressing different CD133 levels. Three different proteic maps were analyzed and 27 spots for each of the two subpopulations were selected for mass spectrometry analysis. They included 3 proteins whose expression appeared constant in all samples and 24 spots that significantly changed (more than two-fold) their intensity. After analysis performed with the dedicated software and searching against the UniProtKB-SwissProt database, only 15 spots were unambiguously identified for both CD133^low^ and CD133^high^ cell populations (Table 1). All the identified proteins were classified on the basis of their main functional role. As shown in Table 2, the majority of proteins that resulted down-modulated in CD133^high^ cells include cell cycle and apoptosis related proteins and proteins involved in actin reorganization. In CD133^high^ cells, 3 proteins were up-regulated, namely the actin-binding protein Tropomyosin 4 (Tm4), the regulator of protein methylation Adenosylhomocysteinase (AdoHcyase) and the Eukaryotic translation initiation factor 3 subunit 2 (eIF3β) (Table 2). The validation of proteomic results was performed by Western blot analysis of whole lysates from CD133^low^ and CD133^high^ cells with antibodies commercially available and of proven specificity directed against some differentially expressed proteins. In particular, the higher amount of Tm4, eIF3β and AdoHcyase and the lower amount of 14-3-3ϵ in CD133^high^ in comparison with CD133^low^ cells was confirmed (Figure 3).

**Table 1:** **Proteins differentially expressed in CD133**^**low **^**and CD133**^**high **^**cells unambiguously identified after two**-**dimensional electrophoresis combined with mass spectrometry**

**Gene symbol**	**Protein name**	**Swiss Prot. Ac. n**°	**Score**	**Coverage (%)**	**Peptides**	**MWt**	**Err ppm**
TPM4	Tropomyosin alpha-4 chain (Tm4)	P67936	651	33	10	29	6
YWHAE	14-3-3 protein epsilon (14-3-3 ϵ)	P62258	116	22	2	29	7
SFN	14-3-3 protein sigma (14-3-3 σ)	P31947	103	13	2	28	9
YWHAB	14-3-3 protein beta/alpha (14-3-3 β/α)	P31946	433	35	9	28	8
TCP1	T-complexprotein 1 subunit alpha (TCP-1-α)	P17987	573	23	9	61	11
SERPINB1	Leukocyteelastaseinhibitor (LEI)	P30740	547	38	10	43	8
SAHH	Adenosylhomocysteinase (AdoHcyase)	P23526	453	19	9	48	7
EIF2S1	Eukaryotic translation initiation factor 2 subunit 1(eIF-2A)	P05198	196	13	4	36	5
PPP2CB	Ser/threo-protein phosphatase 2A cat. sub. betaisof.(PP2A-β)	P62714	125	22	2	36	4
EIF3S2	Eukaryotic translation initiation factor 3 subunit 2 (eIF3β)	Q13347	227	29	3	37	8
DDAH1	NG-dimethylargininedimethylaminohydrolase 1 (DDAH-1)	O94760	274	34	4	31.4	4
PPA1	Inorganic pyrophosphatase (PPase)	Q15181	281	30	6	33	8
ANXA3	Annexin A3	P12429	549	34	10	36.5	4
CAPZB	F-actin capping protein subunit beta (CapZβ)	P47756	351	30	6	31.6	7
PSME3	Proteasome activator complex subunit 3 (PA28γ)	P61289	122	11	3	30	3

**Table 2:** **Biological functions and roles in breast cancer of proteins differentially expressed in CD133**^**low **^**and CD133**^**high **^**cells**

**Proteins**	**Main function/Biological process**	**Known roles in breast cancer**	**CD133**^**high **^**versus CD133**^**low**^
Tm4	Actin-binding protein/Cell motility	Metastasis (32), Chemotherapy resistance (34)	Up
14-3-3 ϵ	Protein binding/Cell cycle	Chemotherapy resistance (34)	Down
14-3-3 σ	Protein binding/Cell cycle, apoptosis	Tumor suppression (35)	Down
14-3-3 β/α	Protein binding/Cell cycle, apoptosis	Chemotherapy resistance (34)	Down
TCP-1-α	Molecular chaperone/Cell motility	Metastasis (36)	Down
LEI	Protease inhibitor/Protein metabolism	Cell migration and invasiveness suppression (37)	Down
AdoHcyase	Hydrolase/Protein metabolism	Oncogenic (38)	Up
eIF-2A	Initiation factor/Protein metabolism	Protein biosynthesis (39)	Down
PP2A- β	Protein phosphatase/Apoptosis	Tumor suppression (40)	Down
eIF3β	Initiation factor/Protein metabolism	Oncogenic (41)	Up
DDAH-1	Hydrolase/Metabolic pathways	Unknown	Down
PPase	Pyrophosphate hydrolysis/Metabolic pathways	Chemotherapy resistance (34)	Down
Annexin A3	Phospholipase A2 inhibitor/Metabolic pathways	Chemotherapy resistance (34)	Down
CapZ β	Actin capping/Cell motility	Chemotherapy resistance (34)	Down
PA28γ	Protein binding/Cell cycle, apoptosis	Unknown	Down

### PLC-β2 promotes the CD133^high^ to CD133^low^ conversion

**Figure 4:**
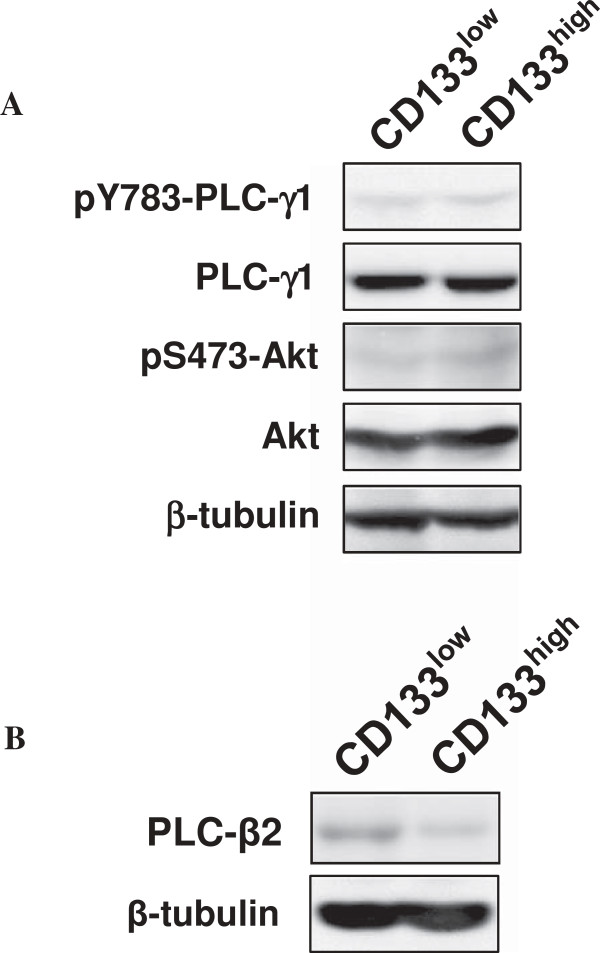
**Signalling molecules in CD133**^**low **^**and CD133**^**high **^**cells. (A)** Immunochemical analysis with the indicated antibodies of total lysates from CD133^low^ and CD133^high^ cells. Tubulin was blotted as a control for the equivalence of loaded protein. **(B)** Western blot analysis of PLC-β2 of immunoprecipitates with an anti-PLC-β2 antibody from CD133^low^ and CD133^high^ cells. Lysates from the same cells were analyzed for β-tubulin content, as internal control of processed proteins. The data are representative of three separate experiments.

To assess if the difference in malignancy-related features between CD133^low^ and CD133^high^ cells may depend to different expression/activation levels of proteins thought to play a role in proliferation and invasiveness of breast tumor cells, a Western blot analysis with specific antibodies was performed. We focused on Akt, PLC-γ1 and PLC-β2, which have been reported to be involved in breast cancer progression, distant metastasis and poor outcome, respectively [18-21]. As shown in Figure 4A, by comparing total lysates from CD133^low^ and CD133^high^ cells, no difference in expression and in levels of phosphorylation of Akt was found. Similarly, PLC-γ1 was expressed at the same level and phosphorylated to the same extent in the two cellular subsets, while the amount of PLC-β2 in CD133^low^ cells was found remarkably higher than in CD133^high^ cells (Figure 4B).

**Figure 5:**
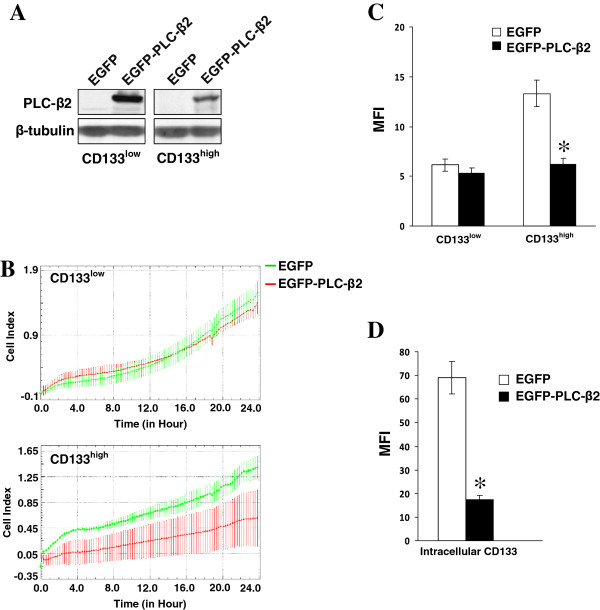
**PLC**-**β2 and CD133 expression. (A)** Western blot analysis with the indicated antibodies of CD133^low^ and CD133^high^ cells transfected with a construct expressing EGFP-tagged human PLC-β2 (EGFP-PLC-β2). **(B)** Dynamic monitoring of invasion through Matrigel using the xCELLigence system of transfected cells. **(C)** Cytofluorimetrical analysis of surface expression of CD133 by direct staining with PE-conjugated 293C3 antibody. **(D)** Cytofluorimetrical analysis of intracellular CD133 with PE-conjugated 293C3 antibody of transfected CD133^high^ cells. Only EGFP-expressing cells were analyzed. The asterisks indicate statistically significant differences (*P* < 0.05). Error bars indicate ± SD. All the data are representative of three separate experiments.

To elucidate whether PLC-β2 may contribute to the different features of cells expressing different CD133 levels, an EGFP-tagged human protein was over-expressed in both CD133^low^ and CD133^high^ cells (Figure 5A). As shown in Figure 5B, the forced expression of PLC-β2 was unable to modify the invasive properties of CD133^low^ cells but induced a significant decrease of invasive potential of CD133^high^ cells. The co-expression of EGFP with PLC-β2 allowed to selectively monitor CD133 in transfected cells, revealing that CD133^high^ cells in which PLC-β2 resulted over-expressed showed a significant reduction of CD133 levels, both at membrane (Figure 5C) and intracellular (Figure 5D).

**Figure 6:**
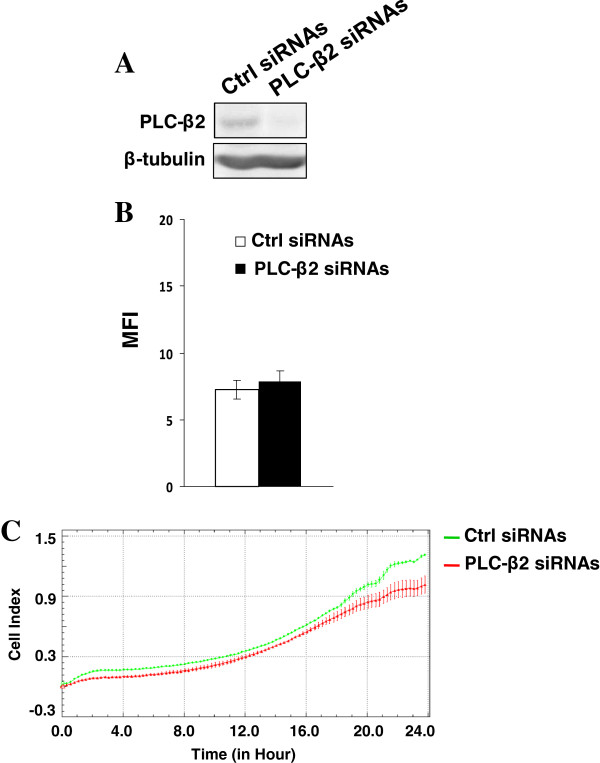
**PLC**-**β2 and invasiveness of CD133**^**low **^**cells. (A)** Immunochemical analysis of immunoprecipitates with the anti-PLC-β2 antibody from CD133^low^ cells transfected with siRNAs specific for PLC-β2 (PLC-β2 siRNAs). A non-silencing scramble siRNAs was used as a control (ctrl siRNAs). Lysates from the same cells were analyzed for β-tubulin content, as internal control of processed proteins. **(B)** Cytofluorimetrical analysis of surface expression of CD133 by direct staining with PE-conjugated 293C3 antibody and **(C)** dynamic monitoring of invasion through Matrigel using the xCELLigence system of transfected cells. Error bars indicate ± SD. All the data are representative of three separate experiments.

Experiments in which PLC-β2 expression in CD133^low^ cells was inhibited with specific siRNAs (Figure 6A) failed to show any modification of CD133 levels (Figure 6B) but evidenced a significant reduction of invasion capability (Figure 6C).

**Figure 7:**
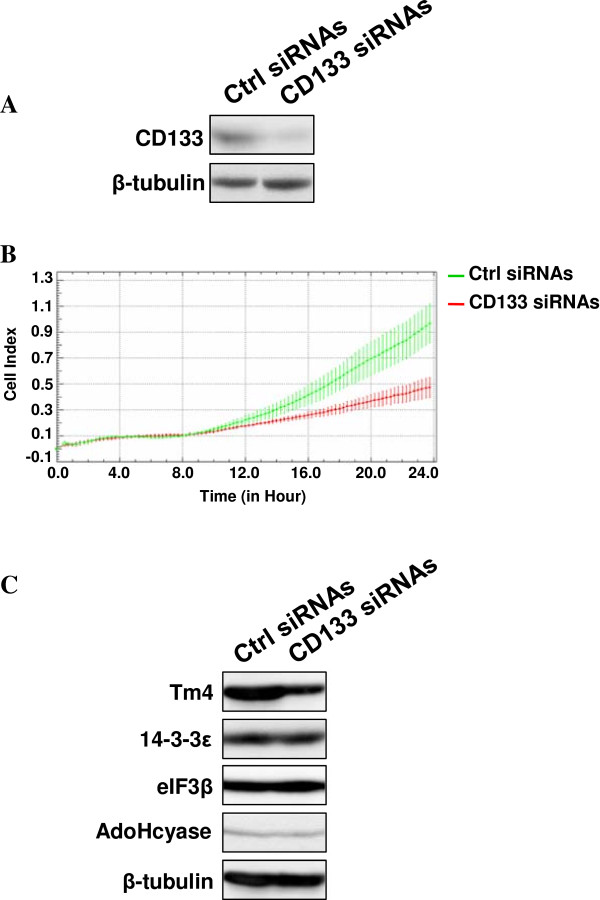
**CD133 and invasiveness of CD133**^**high **^**cells. (A)** Western blot analysis with the anti-CD133 antibody of mmunoprecipitates with anti-CD133 antibody from CD133^high^ cells transfected with specific siRNAs (CD133 siRNAs). A non-silencing scramble siRNAs was used as a control (ctrl siRNAs). Lysates from the same cells were analyzed for β-tubulin content, as internal control of processed proteins. **(B)** Dynamic monitoring of invasion through Matrigel using the xCELLigence system of transfected cells. Error bars indicate ± SD. **(C)** Immunochemical analysis with the indicated antibodies of lysates from CD133^high^ cells in which CD133 expression was down-modulated. Tubulin was blotted as a loading control. The data are representative of three separate experiments.

Down modulation experiments with siRNAs specific for CD133 (Figure 7A) demonstrated that this protein may be involved in determining the high invasive potential of CD133^high^ cells, as shown by the significant decrease of the invasion capability of CD133-silenced cells (Figure 7B). Remarkably, among the proteins differentially expressed in CD133^low^ and CD133^high^ cells, the silencing of CD133 in CD133^high^ cells decreased the expression of Tm4 (Figure 7C), whose elevated amounts have already been correlated with the ability to metastasize of breast tumors [22].

**Figure 8:**
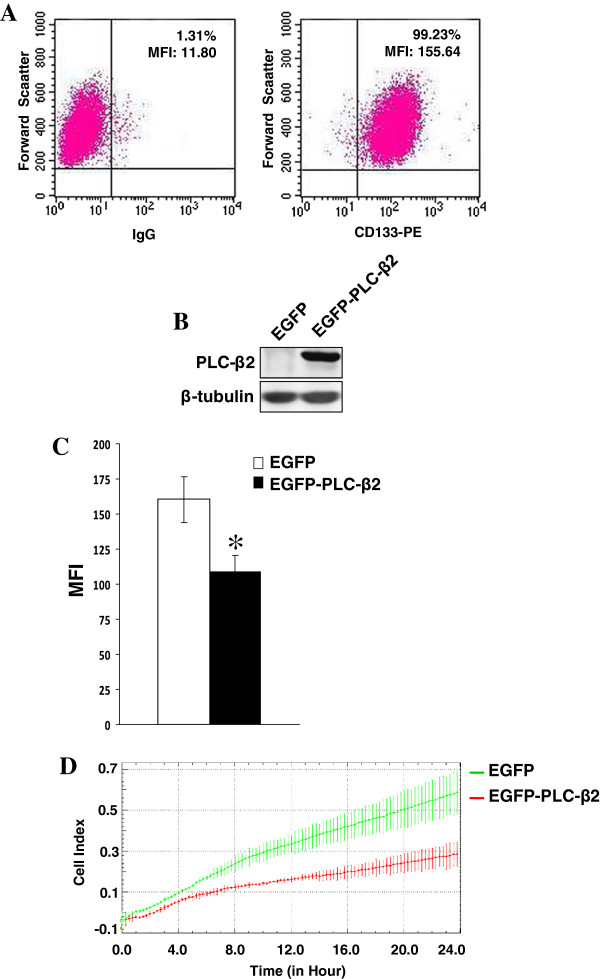
**CD133 and PLC**-**β2 expression in MDA**-**MB**-**468 cells. (A)** CD133 surface expression evaluated in MDA-MB-468 cells by means of flow cytometry after staining with PE-conjugated 293C3 antibody and presented on a biparametric dot plot. The quadrants are gated to separate positive populations. The percentage of cells expressing high levels of CD133 is indicated at the upper right of each panel, together with their mean fluorescence intensity (MFI). **(B)** Western blot analysis with the indicated antibodies of MDA-MB-468 cells transfected with a construct expressing EGFP-tagged human PLC-β2 (EGFP-PLC-β2). **(C)** Cytofluorimetrical analysis of surface expression of CD133 by direct staining with PE-conjugated 293C3 antibody of transfected cells. Only EGFP-expressing cells were analyzed. The asterisk indicates statistically significant differences (*P* < 0.05). **(D)** Dynamic monitoring of invasion through Matrigel using the xCELLigence system of transfected cells. Error bars indicate ± SD. All the data are representative of three separate experiments.

The results indicating that, in triple negative breast tumor cells expressing CD133, the up-regulation of PLC-β2 levels reduces both CD133 expression and invasion capability were confirmed in MDA-MB-468 cells (Figure 8A). In this cell line, in which almost the entire population expresses CD133, the over-expression of PLC-β2, nearly absent in control cells (Figure 8B), significantly reduces CD133 levels (Figure 8C) and the invasion capability (Figure 8D).

## Discussion

Initially considered a marker of hematopoietic stem cells, CD133/prominin is a glycosylated trans-membrane protein expressed in various solid tumors, including breast cancer, in which CD133-positivity seems to identify a restricted subgroup of tumor progenitors [23,24]. In normal mammary tissue, CD133/prominin is not a marker for stem cells and seems to regulate ductal branching [25]. Beyond its possible relationship with stemness of tumor cells, CD133 expression in breast cancer significantly correlates with tumor stage, tumor size and occurrence of lymph node metastases [26]. CD133 is also useful in predicting chemosensitivity to neoadjuvant chemotherapy in breast cancer [27], suggesting that CD133 expression may be of help in more accurately predicting the aggressive properties and in determining the optimal therapeutic strategy for this neoplasia. A strong correlation of CD133 expression with clinical stage of breast tumor patients was observed in TNBC (ER-, PR- HER2-), a high risk breast neoplasia that lacks the benefit of specific therapy that targets these receptors [3]. It has been recently demonstrated that the expression of CD133 is associated with markers of hypoxia and/or tumor microvasculature in human breast tumors [12,28] and, in TNBC, CD133(+) cells with cancer stem cell characteristics associate with vasculogenic mimicry [29]. These data suggest that the tumor microenvironment, and in particular hypoxia, induces in breast cancer cells a basal-like phenotype that includes increased expression of CD133 and decreased expression of hormone receptors.

CD133 is expressed at the surface of several cancer cells, not only with characteristics of stemness [30], but a direct function of CD133 in determining specific features of tumor cells was not described. In particular, nothing is known about the role of CD133 in determining the biological properties of TNBC cells. This issue was tentatively addressed with the highly tumorigenic and moderately metastatic MDA-MB-231 cells [31], which show an ER-, PR-, Her2- immunoprofile, mimicking the condition that is characterized by a low response to chemotherapy and worst prognosis in breast tumor patients [32]. Here we show that the cytofluorimetrical analysis with anti CD133 antibodies identifies, in the bulk of the cell population, a low basal CD133 expression, and in a small percentage of cells (2-3%), a much higher expression level, making this cell line useful to compare TNBC cells with different levels of CD133 expression. By using antibodies directed against different CD133 epitopes [33,34] and Tunicamycin we ruled out the potential bias arising from variable glycosylation levels and from glycosylation-dependent epitopes in the extracellular portion of CD133 that it was reported to be potentially lost upon differentiation of tumor cells [5]. We also extended the analysis to intracellular CD133 that allowed to definitely confirm the existence, in MDA-MB-231 cells, of a small but stable subpopulation expressing high levels of CD133 in both membrane and cytoplasm compartments. A comparison between cells expressing either low or high levels of CD133 indicates that CD133^high^ cells show lower proliferation and migration rate together with a larger adhesion area, consistent with a more undifferentiated tumoral phenotype. Interestingly, CD133^high^ cells exhibit a higher invasion capability through Matrigel, suggestive of higher metastatic potential. This is consistent with the data obtained in triple negative tumors, in which CD133 expression levels positively correlate with metastatization to lymph nodes [3].

Protein profiles of CD133^low^ and CD133^high^ cells were compared by means of 2D analysis followed by mass spectrometry, showing that a number of proteins already known to be de-regulated in breast cancer [22,35-42] are differentially expressed between the two sub-populations. In particular, CD133^low^ cells that proliferate and migrate faster than CD133^high^ cells, show higher expression of proteins regulating cell motility. Interestingly, CD133^high^ cells, which exhibit a more invasive phenotype, show higher expression of the actin-binding protein Tm4, that was reported to be up-regulated in highly metastatic breast cancer cell lines and to be associated with the presence of lymph node metastasis of breast tumors [22]. Tms are a family of cytoskeletal proteins present in virtually all eukaryotic cells, where they bind actin filaments and stabilize their structure [43]. Changes in the expression of specific Tms are commonly found in malignantly transformed cells and overexpression of Tm4 in breast cancer cells is related to metastatic behaviour and may be a useful marker for predicting distant metastasis [32]. In comparison to CD133^low^ cells, CD133^high^ cells also express higher levels of AdoHcyase, known to play a key role in the control of methylation [44] and that, in breast cancer, seems to be involved in regulation of histone methylation via the 2 member enhancer of zeste homolog 2 (EZH2) [39]. Since inhibition of AdoHcyase results in G2/M cell cycle arrest, apoptosis and cellular differentiation of breast tumor cells, including MDA-MB-231 [39], targeting of this enzyme might be of therapeutic value in breast cancer.

Also the expression levels of a member of the eukaryotic initiation factor eIF3 family is higher in CD133^high^ than in CD133^low^ cells. eIF3 complex is essential for initiation of protein synthesis and the β subunit was already reported to be over-expressed in human breast carcinoma [42]. Data on glioblastoma cells suggested for eIF3β an oncogenic role since its down-modulation inhibited cell proliferation and increased the apoptosis rate [45]. This evidence indicates that, at least in TNBC cells, high expression of CD133 identifies cells with a peculiar protein expression pattern which accounts for their relatively differentiated tumoral phenotype together with high metastatic potential. Concerning the signalling molecules known to modulate proliferation/motility of breast tumor cells, no differences have been observed between CD133^high^ and CD133^low^ cells in the expression and activation levels of Akt, whose activity seems to have dichotomous effects on neoplastic progression of breast cancer [19]. Also expression and activation levels of PLC-γ1, correlated with distant metastases of early breast tumors [21] and involved in metastatic properties of TNBC cells [46] were investigated. However, no difference between the two sub-populations expressing different levels of CD133 was found. On the contrary, CD133^high^ cells express PLC-β2 at levels significantly lower than CD133^low^ cells, in accordance with our previous data indicating that, in breast tumor-derived cells, PLC-β2 amount positively correlates with proliferation rate and motility [16]. In particular, our previous studies on MDA-MB-231 cells, 98% of which express basal levels of CD133, have demonstrated that the down-modulation or the over-expression of PLC-β2 respectively reduces or increases their proliferation and migration rate [16]. On the other hand, we have demonstrated that the silencing of PLC-β2 decreases invasion capability of MDA-MB-231 but its overexpression fails to affect their invasion capability through Matrigel [16], indicating that the sole PLC-β2 is necessary but not sufficient to sustain the metastatic potential of TNBC cells. Here we show a peculiar role of PLC-β2 in cells expressing high levels of CD133. In fact, the over-expression of PLC-β2 in CD133^high^ cells, which contain relatively low levels of the protein, is able to induce a significant decrease of their invasion capability, in parallel with a reduced expression of CD133, at both membrane and cytoplasm levels. When the expression of PLC-β2 was down-modulated in CD133^low^ cells, containing relatively high levels of the protein if compared with CD133^high^ cells, a significant decrease of invasion capability was observed, according with our data previously obtained on the entire MDA-MB-231 cell population (accounting for about 98% of CD133^low^ cells) [16]. The lack of effects of PLC-β2 down-modulation on CD133 expression in CD133^low^ cells confirms that the two sub-populations expressing different CD133 levels correspond to different stages of tumor differentiation, in which different signalling mechanisms take place. In this context, while PLC-β2 promotes the conversion of CD133^high^ to CD133^low^ cells, its silencing in cells showing a more differentiated tumoral phenotype (CD133^low^) is not sufficient to revert the phenomenon.

A reduction of invasiveness trough Matrigel of CD133^high^ cells was found also when CD133 expression was forcedly down-modulated by specific siRNAs, indicating that CD133 is primarily involved in invasion capability of TNBC-derived cells. The mechanism may be correlated with the preferential localization of CD133 in plasma membrane protrusions, ended to regulate lipid composition and membrane topology [4]. By establishing and maintaining membrane protrusions, CD133 may be involved in cell polarity and migration and may regulate the invasive properties of TNBC cells. On the other hand, the decreased expression of Tm4 observed after down-modulation of CD133 in highly expressing cells allows to speculate on a more specific mechanism by which CD133 can promote invasiveness of tumor cells, taking into account that the expression of specific isoforms of the Tms family correlates with the metastatic potential of TNBC-derived cells [22].

The results indicating that up-regulation of PLC-β2 in cells expressing high levels of CD133 reduces the expression of this glysosylated protein in parallel with the invasion capability of CD133^high^ cells was confirmed in MDA-MB-468, a triple negative cell line expressing CD133 at high levels [47] and almost negative for PLC-β2. The overall results indicate that, in TNBC cells, the increased expression of PLC-β2 down-regulates invasiveness only in cells with high levels of CD133 since this PLC isozyme negatively modulates the expression of CD133, in turn involved in determining the invasive properties of CD133^high^ cells.

## Conclusions

The high expression of CD133 in TNBC-derived cells correlates with high invasive potential and with a peculiar pattern of protein expression that includes the up-regulation of molecules correlated with lymph node metastasis of breast tumors. The aggressive properties of CD133^high^ cell are mitigated by PLC-β2 which, despite its general role in sustaining motility of breast tumor cells, down-modulates the expression of CD133 and thus may play a role in preventing metastatic progression of CD133 positive TNBC.

Considering that the relevance of CD133 in malignancy of breast tumors is well established, our finding that PLC-β2 is involved in CD133-mediated invasiveness of cells derived from TNBC can contribute to better estimate the prognosis and more accurately identify therapeutic targets for TNBC, which remains a highly heterogeneous type of cancer and often an incurable illness.

## Materials and methods

### Cell culture and reagents

All reagents were from Sigma (St Louis, Mo., USA) unless otherwise indicated.

The breast cancer-derived cell line MDA-MB-231 and MDA-MB-468 and the human colon cancer cell line Caco-2 were purchased from the American Type Culture Collection (Rockville, MD). MDA-MB-231 and MDA-MB-468 cells were grown in high-glucose Dulbecco's modified Eagle's medium (DMEM, Gibco Laboratories, Grand Island, NY) supplemented with 10% fetal bovine serum (FBS, Gibco Laboratories). Caco-2 cells were cultured in DMEM with 1% Non-essential Amino Acid (NEAA, Lonza Sales Ltd, Basel, CH), 1% Sodium Pyruvate (Gibco Laboratories), 1% Penicillin-streptomycin solution (Lonza) and 10% FBS. All cell lines were grown at 37°C in a humidified atmosphere of 5% CO2 in air.

To inhibit N-glycosylation, Caco-2 and MDA-MB-231 cells were cultured in the presence of 2.5 μg/ml Tunicamycin or vehicle (medium containing 0.1% DMSO) for 24 hours.

### Evaluation of CD133 expression

CD133 surface expression was evaluated by means of flow cytometry by direct staining of the cells with phycoerythrin (PE)-conjugated anti-CD133/1 (AC133) and anti-CD133/2 (293C3) mouse monoclonal antibodies (Miltenyi Biotec, Bologna, I), as suggested by manufacter's protocol, and by indirect labelling with a hybridoma supernatant (clone 7) containing a monoclonal antibody directed against unmodified CD133 epitopes, kindly provided by Dr. Panyam and Ohlfest (University of Minnesota) and used as described by Swaminathan et al. [33]. In particular, 5×10^5^ cells were stained with 100 μl of clone 7 hybridoma supernatant and reacted with a secondary anti-mouse-PE antibody (Becton-Dickinson, San José, CA).

For analysis of intracellular amounts of CD133, Perm and Stab Solutions Kit (Instrumentation Laboratory S.p.A, Milan, I) was used, performing the staining with anti-CD133/2-PE antibody, as suggested by manufacturers.

All the samples were analyzed by a FACSCalibur flow cytometer (Becton-Dickinson) with CellQuest Pro 6.0 software (Becton-Dickinson). Data collected from 10 000 cells are shown as percentage of positive cells or as mean fluorescence intensity (MFI) values.

### Immunomagnetic separation

MDA-MB-231 cells were resuspended in PBS containing 0.5% bovine serum albumin and 2 mmol/L EDTA. For magnetic labeling, CD133/1 Micro Beads were used (Miltenyi Biotech) and positive magnetic cell separation was done using MACS SD columns (Miltenyi Biotech), according to manufacturer's instructions. CD133^low^ and CD133^high^ subpopulations were cultured in the same above reported medium and subjected to morphological analysis, to xCELLigence RTCA assays and to modulation of PLC-β2 and CD133 expression.

### Two-dimensional gel electrophoresis and mass spectrometry

2-DE was performed essentially as described by Bertagnolo et al. [48], with some modifications. Briefly, CD133^low^ and CD133 ^high^ cells were lysed with 2 M thiourea, 7 M urea, 4% CHAPS, 1% DTT, 2% IPG buffer pH 3–10 (Bio-Rad, Hercules, CA), benzonase and protease inhibitors, followed by heating for 30 min at 30°C, sonication and centrifugation at 21 000 × *g* for 60 min at 4°C. Supernatant containing 400 *μ*g of proteins was used to rehydrate 17 cm pH 4–7 IPG gel strips (Bio-Rad) for 16 h at 20°C. Focusing was carried out on PROTEAN IEF cell (Bio-Rad) using the following conditions: 250 V (60 min), 500 V (60 min), 1000 V (60 min), 5000 V (60 min), 10 000 V (60 min) and 10 000 V for the additional time required to reach a total of 80 kVh. The separation in the second dimension was performed using 1 mm thick, 12% constant vertical SDS-PAGE in PROTEAN II xi apparatus (Bio-Rad) at constant 35 mA/gel. The gels were stained with Coomassie Brilliant Blue G-250 (Bio-Rad) and scanned using a Pharos-FX Molecular Imager (Bio-Rad). The acquired maps were analyzed using the PDQuest Basic Version 8.0 software (Bio-Rad), as previously reported [48]. A difference in intensity of 200% between spots of two compared gels was considered significant. Spots of interest were excised using a sterile blade and subjected to mass spectrometry analysis essentially as described by Bavelloni et al. [49]. For peptide sequence searching, monoisotopic peptide mass lists were submitted to Mascot v.2.1 (Matrix Science, London, UK) against the UniProtKB-SwissProt database (April 23, 2012, total of 535 698 entries). The search parameters were as follows: two missed cleavage allowed, carbamidomethylation of cysteine as fixed modification, oxidation of methionines as variable modification, precursor ion mass tolerance 50 ppm and fragment ion tolerance 1 Da.

### Analysis of adhesion area

The morphology of CD133^low^ and CD133^high^ MDA-MB-231 cells was analyzed with an inverted phase-contrast microscope (Nikon Eclipse TE2000-E; Nikon, Florence, I) and cell images were acquired by the ACT-1 software with a DXM1200F digital camera (Nikon) and analyzed with the ImageJ software, as previously reported [50].

### Real-time cell proliferation, migration and invasion assays

Cell proliferation, migration and invasiveness were evaluated by means of the xCELLigence RTCA System (Real-Time Cell Analyzer System, Roche Applied Science, Mannheim, D), developed to monitor cell events in real time by measuring the electrical impedance produced by cells. The employed procedures were essentially those described by Stander et al. [51] for proliferation kinetics and by Mandel et al. [52] for migration and invasiveness assays. In particular, to measure cell proliferation, 5000 cells ⁄well were used with a programmed signal detection every 15 min for a total of 96 h. For migration assays, 4 × 10^4^ cells∕well were seeded onto the top chambers of CIM-16 plates (Roche) and the bottom chambers were filled with medium containing 5% serum. The setup for analysis of invasiveness was the same described for migration except that the upper side of the membranes was covered with a layer of Matrigel (BD Biosciences, San Josè, CA) diluted 1:20 and the bottom chambers were filled with 10% serum containing medium. For both migration and invasion assays, the signal detection was programmed every 15 min for a total of 24 h. Impedance values were expressed as a dimensionless parameter (cell index, CI).

### Modulation of PLC-β2 and CD133 expression

PLC-β2 over-expression was performed by transient transfection with a plasmid expressing an Enhanced Green Fluorescent Protein (EGFP)-tagged full-length human PLC-β2, as previously reported [16].

The down-modulation of CD133 and of PLC-β2 was performed by silencing the proteins with specific siRNAs (Santa Cruz Biotechnology, Santa Cruz, CA), following a previously described procedure [16]. As a control of transfection efficiency a non-silencing fluorescein-labeled duplex RNA, purchased from Qiagen (Milan, I), was used. The transfected cells were incubated at 37°C in a 5% CO_2_ atmosphere for 48 h and then subjected to immunochemical and cytofluorimetrical analysis and to xCELLigence RTCA assays.

### Immunoprecipitation and immunochemical analysis

PLC-β2 was immunoprecipitated from CD133^low^ and CD133^high^ MDA-MB-231 cells and CD133 was immunoprecipitated with an anti-CD133/1 (W6B3C1, Miltenyi) from MDA-MB-231, CD133^high^ MDA-MB-231 and Caco-2 cells following a previously reported procedure [16].

Total lysates and immunoprecipitates were separated on 7.5% polyacrylamide denaturing gels and blotted to nitrocellulose membranes (GE Healthcare Life Science, Little Chalfont, UK). The membranes were then incubated with antibodies directed against pY783-PLCγ1, PLC-γ1, PLC-β2, 14-3-3ϵ, eIF3β, AdoHcyase and Akt (Santa Cruz Biotechnology), pS473-Akt and Tm4 (Millipore S.p.A., Milan, I), CD133/1 (W6B3C1, Miltenyi Biotec) and β-tubulin (Sigma). The chemiluminescence derived bands were acquired with ImageQuant™ LAS 4000 biomolecular imager (GE Healthcare) and the densitometric analysis was performed by means of Image Quant TL software (GE Healthcare).

### Statistical analysis

The results were expressed as means ± standard deviations of three independent experiments. Statistical analysis was performed by using the two-tailed Student's t-test for unpaired data. P values ≤0.05 were considered statistically significant.

## Abbreviations

TNBC: Triple-negative breast cancers; ER: Estrogen receptor; PR: Progesterone receptor; PLC: Phosphoinositide-dependent phospholipase C; Tm4: Tropomyosin 4; AdoHcyase: Adenosylhomocysteinase; eIF3β: Eukaryotic translation initiation factor 3 subunit 2.

## Competing interests

The authors declare that they have no competing interests.

## Authors’ contributions

FB and VB participated in the design of the overall study. FB carried out the cytofluorimetrical and Real-time cell analysis. SG performed mono-dimensional and bi-dimensional protein analysis. MP^1^ and EN carried out transfection experiments. MP^2^ and AB performed mass spectra analysis. FB and VB conceived the study and interpreted the results. VB and SC wrote the paper and all authors read and approved the final manuscript.

## Supplementary Material

Additional file 1: Figure S1 Total lysates from Caco-2 and MDA-MB-231 cells, cultured in the presence of 2.5 μg/ml Tunicamycin or vehicle (DMSO) for 24 hours, were immunoprecipitated with the W6B3C1 anti-CD133 antibody and subjected to Western blot analysis. The data are representative of two separate experiments performed in duplicate. Click here for file
