# Supplementary figures and images for: In triple negative breast tumor cells, PLC-β2 promotes the conversion of CD133high to CD133low phenotype and reduces the CD133-related invasiveness
Source: Mol Cancer. 2013 Dec 13;12:165. doi: 10.1186/1476-4598-12-165 (PMC3866498; doi:10.1186/1476-4598-12-165)

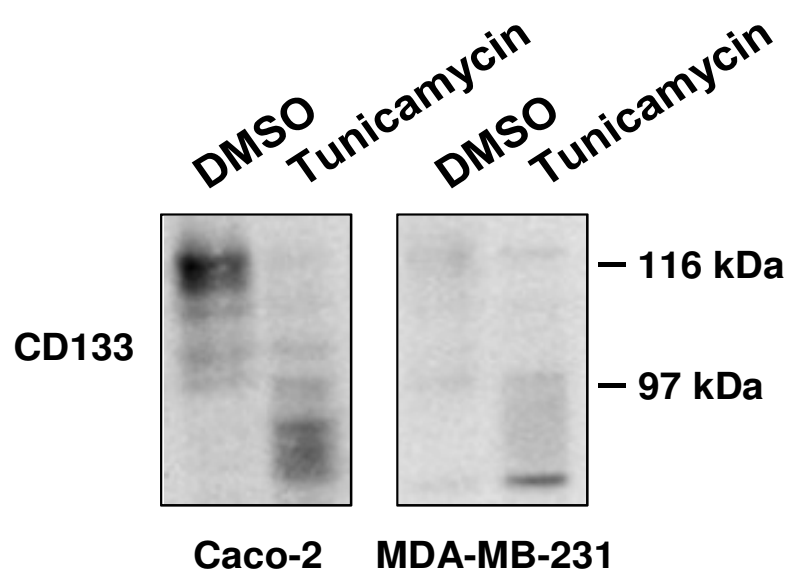

**SupplementaryFIGURE 1**

Supplement: Additional file 1: Figure S1 — Total lysates from Caco-2 and MDA-MB-231 cells, cultured in the presence of 2.5 μg/ml Tunicamycin or vehicle (DMSO) for 24 hours, were immunoprecipitated with the W6B3C1 anti-CD133 antibody and subjected to Western blot analysis. The data are representative of two separate experiments performed in duplicate. [file 1476-4598-12-165-S1.pdf]
